# Cross‐national comparison of factors related to stressors, burnout and turnover among nurses in developed and developing countries

**DOI:** 10.1002/nop2.1002

**Published:** 2021-07-26

**Authors:** Takashi Ohue, Supaporn Aryamuang, Laura Bourdeanu, Jean N. Church, Hamidah Hassan, Jaruwan Kownaklai, Arlene Pericak, Amorn Suwannimitr

**Affiliations:** ^1^ Department of Nursing Faculty of Nursing Hyogo University Kakogawa Japan; ^2^ Department of Nursing Mahasarakham University Mahasarakham Thailand; ^3^ School of Nursing Excelsior College Albany NY USA; ^4^ Faculty of Health Kwantlen Polytechnic University Vancouver Canada; ^5^ Faculty of Medicine and Health Sciences University Malaysia Sabah Kota Kinabalu Malaysia; ^6^ The George Washington University Washington DC USA

**Keywords:** burnout, International comparison, nurse, nurse shortages, stressor, turnover

## Abstract

**Aim:**

To examine factors of a hypothetical model related to stressors, burnout and turnover in nurses from developed and developing countries—Canada, Japan, the United States, Malaysia and Thailand.

**Design:**

A cross‐sectional questionnaire‐based study.

**Methods:**

Conducted between April 2016 and October 2017, the Maslach Burnout Inventory, Intention to Leave Scale, and Nursing Stress Scale collected data from acute care hospital nurses in Canada (*n* = 309), Japan (*n* = 319), Malaysia (*n* = 242), Thailand (*n* = 211) and the United States (*n* = 194).

**Results:**

Compared to other countries, burnout “exhaustion” was the highest in Japan and “cynicism” and intention to leave the job were the highest in Malaysia. Thailand had lower burnouts and turnover than other countries and higher professional efficacy than Japan and Malaysia. In all countries, reducing stressors is important for reducing burnout and intention to leave jobs, especially as they relate to “lack of support.”

## 1 INTRODUCTION

The World Health Organization (WHO, 2006) estimates that the world's current 59 million healthcare workers represent a shortage of 4.3 million workers. This problem is particularly significant in Canada and the United States (US). The Canadian Nurses Association (CNA, 2009) suggests that, if Canadians’ health needs continue along the same path, there could be a dearth of almost 60,000 registered nurses by 2022. The Canadian Institute for Health Information (2015) reported that the number of regulated nurses declined by 0.3% between 2013 and 2014. As of 2012, it was predicted that 64% of Canada's employed nurses could retire within the next 15 years at age 55, while in the United States, the Bureau of Labor Statistics (2012) predicted that 11 million additional nurses will need to be added to the workforce to avoid projected shortages.

These countries are struggling to educate enough nurses within their borders and are offsetting their shortages through the immigration of internationally educated nurses, primarily from developing countries. In Canada, for example, such applicants to regulatory bodies almost tripled from 1999 to 2003 (Jeans et al., 2005).

Japan, which could face a shortage of up to 270,000 nurses by 2025 (Ministry of Health, Labour and Welfare, 2019a), has started to accept nurses and caregivers from countries such as Indonesia and the Philippines under its Economic Partnership Agreement. Globally, one in three healthcare workers is employed in North America, and 40% of nurses employed in Canada, the United States and England are from foreign countries. The United States employs the most foreign nurses (337,000), followed by the UK (82,000), Canada (49,000) and Australia (47,000) (Organisation for Economic Cooperation & Development, 2007). This means that there now exists an unbalanced structure, whereby the rest of the world is covering the nursing shortages in developed countries. The current and predicted nursing shortage compounds the demand for nurses. The average ratio of nurses to the general population in Europe is 10 times that of Southeast Asia (Drennan & Ross, 2019). Further, the WHO report on the healthcare workforce identified 57 countries with critical workforce shortages, six of which were in Southeast Asia (Kanchanachitra et al., 2011). Woo et al., (2020) conducted a systematic review of the global literature on nurses’ burnout and found the highest levels—13.68%—in the Southeast Asian and Pacific region. North America, which includes Canada and the United States, accounts for only 10% of the global disease burden. Nevertheless, almost 37% of the world's healthcare workers live and work in the region, and this region spends more than 50% of the world's financial resources on health. In contrast, Southeast Asia bears more than 30% of the global burden of disease, but is home to only 12% of the world's healthcare workforce (WHO, 2006). The shortage of nurses in Southeast Asia is also serious. For example, in Malaysia, the total number of nursing personnel is 79,700 (Siew et al., 2011) and the turnover rate increased by more than 50% between 2005 and 2010. In 2005, 400 nurses quit their jobs; and in 2010, 1,049 withdrew. Malaysia also faced a migration problem of 400 nurses per year leaving the country to work elsewhere. Currently, approximately 25,000 Malaysian nurses are working in other countries, including the UK and the United States (Barnett et al., 2010; Siew et al., 2011). According to data from the Thailand Nursing and Midwifery Council (TNC) from 2010 to 2016, a nurse‐to‐population ratio of 1:400 in Thailand requires between 163,500 and 170,000 nurses (Khunthar, 2014; Srisuphan & Sawaengdee, 2012). However, in 2010, only 130,388 nurses provided health care services in Thailand's healthcare facilities; during that period, both the public and private sectors therefore faced a shortage of 33,112 nurses (Khunthar, 2014).

The 2030 Agenda for Sustainable Development, formulated by the United Nations goals of 17 Sustainable Development Goals (SDGs), has 169 targets that address the social, economic and environmental determinants of health. The SDGs are organized into five main thematic areas—People, Planet, Peace, Prosperity and Partnership—and aim to build a just, peaceful and inclusive society. However, this agenda is not legally binding, and countries prioritize the SDGs and targets that are relevant to them. The primary focus of the SDGs is to “leave no one behind.” Given that nurses are the primary providers of healthcare across different settings, it is important to elaborate on this idea in the context of how nurses should work on a community‐wide health strategy (United Nations Development Programme, 2018). Goal three is to “ensure healthy lives and promote well‐being of all ages.” This is a more direct link to nurses and nursing care.

The goal of inclusivity will not be achieved if governments and world leaders do not invest in the nursing profession. This has been particularly problematic in developing nations, where hospitals are short‐staffed and medical/nursing care is insufficient. Therefore, achieving the SDGs requires procuring more nurses in developing nations and increasing the quality of nursing services.

## 2 BACKGROUND

Research on burnout syndrome in hospital nurses shows that stressors in the work environment are an important component that contributes to shortages in the workforce, as nurses voluntarily leave their jobs (Applebaum et al., 2010). Thus, the relationship between burnout and turnover is an established one (Ohue et al., 2011; Woo et al., 2020).

The WHO has validated the concept of burnout in the workplace by identifying it as a potential medical diagnosis, which has been included in the 11th version of the International Classification of Diseases. Generally, burnout is defined as “a syndrome of mental/physical fatigue and emotional exhaustion that occurs among individuals who work with people in a certain capacity over prolonged periods, and also experience self‐abasement, distaste for work and lack of compassion for others” (Maslach & Jackson, 1981). Although it was initially restricted to the helping professions, it was later broadened and defined as a crisis in one's relationship with work in general and not necessarily as a crisis in one's relationship with people at work (Maslach et al., 2001). The original three burnout dimensions were redefined and an alternative version of the MBI‐General Survey (MBI‐GS) was developed, which can also be used outside human services occupations (Schaufeli et al., 1996). In other words, exhaustion—as operationalized in MBI‐GS—refers to severe malaise regardless of its cause; cynicism reflects an indifferent or distant attitude towards one's work, rather than others; and lack of professional efficacy includes both the social and non‐social aspects of professional achievement. For a diagnosis based on WHO criteria, three aspects are significant: energy depletion or exhaustion, increased mental distance from work or feelings of work‐related negativism or cynicism, and decreased professional effectiveness (Prior, 2019).

More than 40% of hospital staff nurses score in the high range of work‐related burnout, and more than one in five hospital staff nurses report that they intend to leave within a year (Aiken et al., 2002).

To date, studies have compared physical and mental health (Lambert et al., 2004), but there are no comparative studies on the various factors related to nurse burnout and turnover between developed and developing nations. Herein, we examined the characteristics and associated factors of nurse burnout and turnover in Japan and other countries. While other studies have brought the issues of nurse burnout and turnover to the world's attention, the originality of the current study lies in its international comparison, which is unique in the literature. Individual countries have implemented measures for nurse stressors, burnout and turnover; however, nurse shortages are a global problem. Moreover, nurses from developing nations are migrating to developed nations, which may have a significant effect on the world's ability to achieve the SDGs.

The purpose of this study is to examine factors related to stressors, burnout and turnover among nurses from developed and developing countries. To this end, we compared the developed nations of Japan, the United States and Canada to the developing nations of Thailand and Malaysia in terms of nurse burnout and turnover. We also examined a hypothetical model in which stressors, burnout and turnover were introduced.

## 3 THE STUDY

### 3.1 Design

**FIGURE 1:**
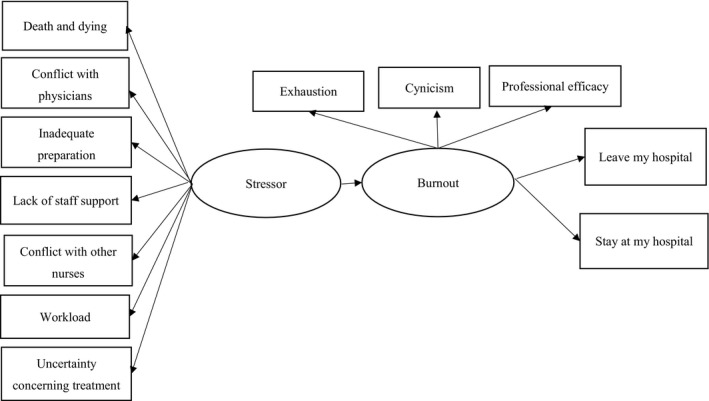
Hypothetical model of this study

A cross‐sectional questionnaire survey was conducted, and a hypothetical model was constructed (Figure 1) with reference to the model of burnout developed by Maslach et al. (1996), which emphasizes the influence of personal stressors or demands and the lack of personal and environmental resources in the development of burnout symptoms. Maslach et al. (1996) list work overload and personal conflict as specific “demands” that contribute to burnout. Coping ability, social support, autonomy and decision involvement are specified as resources, which, when insufficient, contribute to burnout. Characterized by exhaustion, cynicism and a decreased professional efficacy, burnout can lead to a variety of negative outcomes, including diminished organizational commitment, increased job turnover and absenteeism and/or physical illness.

Based on the above, a hypothetical model of “stressor ⇒ burnout ⇒ turnover intention” was examined, with reference to Maslach et al. (1996) model (Figure 1). If this model is verified, it is possible that the reduction of stressors in each country can lead to a reduction in burnout and turnover.

### 3.2 Method

#### 3.2.1 Sample

Convenience sampling was used to obtain the sample. Participants were nurses working in acute care hospitals in Canada, Japan, Malaysia, Thailand and the United States. A survey was conducted in three hospitals in each country.

### 3.3 Instrument

The data were collected using a self‐report questionnaire‐based survey between April 2016 and October 2017 in the five countries. The response rate was 40%–60% in each country. The details of the survey and measurement scales are as follows.

1. Demographic information: We focussed on 11 variables to gather demographic information: gender, age, marital status, academic background, position at work, type of employment (full‐time, part‐time, contract), number of employees in the workplace, frequency of exercise, frequency of participating in hobbies or leisure activities, time absent from work over the last year due to health reasons and amount of overtime per month.
2. Stressor measurement: We used the Nursing Stress Scale (NSS; Funashima et al., 1997; Gray‐Toft & Anderson, 1981), which is the best‐known and most widely used tool for assessing work‐related stress in this population. Gray‐Toft and Anderson (1981) developed this instrument to assess the frequency and major causes of stress as perceived by hospital nurses. The instrument consists of 34 items describing situations identified as causing stress to nurses while performing their duties. It provides a total stress score and a score for each of the seven subscales measuring the *frequency* of stress experienced by nurses in the hospital environment (Gray‐Toft & Anderson, 1981). The seven subscales are as follows: “death and dying” (seven items), “conflict with physicians” (five items), “inadequate preparation” (three items), “lack of support” (five items), “conflict with other nurses” (five items), “workload” (six items) and “uncertainty concerning treatment” (five items).
3. Burnout measurement: Usually, the Maslach Burnout Inventory‐Human Services Survey is used for nurses. However, stable results have not been observed, such as support for the four‐factor structure in the Japanese version (Kitaoka−Higashiguchi, 2004). Thus, we used the Maslach Burnout Inventory‐General Survey (MBI‐GS; Kitaoka‐Higashiguchi et al., 2004; Schaufeli et al., 1996). The MBI‐GS was designed for use with occupational groups other than human services and education, including those working in jobs such as customer service, maintenance, manufacturing, management and most other professions. The MBI‐GS contains three scales: “exhaustion” (EX: five items), “cynicism” (CY: five items) and “professional efficacy” (PE: six items). The EX scale measures feelings of being overextended and tired out by one’s work; CY measures indifference or a distant attitude towards one’s work; and PE measures satisfaction with past and present accomplishments. It explicitly assesses an individual’s expectations of continued effectiveness at work. All items are scored on a seven‐point Likert scale ranging from 0 (“never”) to 6 (“every day”). High EX and CY scores and low PE scores are indicative of burnout.
4. Intention to leave: Intention to leave was measured with the “intent to leave or stay” subscale of Kim et al., (1996) Career Intent Scale. The scale consists of two items measured on a five‐point Likert scale from 1 (“not at all”) to 5 (“very much”). A higher total score indicates a greater intention to leave. The Intention to Leave Subscale contains two further subscales: “I plan to leave my hospital as soon as possible” and “I plan to stay in my hospital as long as possible.”
5. Comments: We asked the respondents to describe their stress and reasons for wanting to quit nursing in the comments section.

For nurses in Japan, we created materials in Japanese. For nurses in the United States, Canada, Thailand and Malaysia, we developed the materials in English. We obtained approval for distributing the questionnaires from the responsible parties at the participating hospitals. Collaborative researchers from each country negotiated with regional hospitals and mailed a questionnaire to those that provided their consent.

### 3.4 Validity, reliability and rigour

The NSS, MBI‐GS and Intention to Leave Scale were selected as the parameters of this study. The internal consistency of the NSS was measured by Cronbach's alpha and had a value of 0.89 (Gray‐Toft & Anderson, 1981). In addition, for the Japanese version of the NSS (Funashima et al., 1997), Cronbach's alpha was 0.91. The results of the NSS factor analysis were similar to the results of the original version, with small differences. The validity of the NSS was supported by statistical associations with trait anxiety, state anxiety, job satisfaction and turnover, which are theoretically related to work stressors in nurses (Gray‐Toft & Anderson, 1981).

Thereafter, the MBI‐GS was used (Kitaoka‐Higashiguchi et al., 2004; Schaufeli et al., 1996). Leiter and Schaufeli (1996) found the internal consistency of each of the instrument's subscales to be satisfactory; Cronbach's alpha coefficients ranged from 0.84 to 0.90 for EX, 0.74 to 0.84 for CY and 0.70 to 0.78 for PE. Additionally, when the Japanese MBI‐GS was administered to a sample of hospital workers, exploratory factor analysis revealed three factors similar to the original MBI‐GS (Kitaoka‐Higashiguchi et al., 2004).

We employed the Intention to Leave Scale (Kim et al., 1996), used by Kovner et al., (2009) and Brewer et al. (2012) to assess newly hired registered nurses’ intent to stay.

These tools have provided satisfactory results with similar populations worldwide, supporting their use in the current study.

### 3.5 Analysis

1. We conducted a one‐factor analysis of variance for nurses working in hospitals in the United States, Canada, Japan, Thailand and Malaysia using stressors, burnout and intention to leave. The Tukey method was used for multiple comparisons.
2. We examined the hypothetical model depicted in Figure 1, in which stressors, burnout and turnover were introduced. The model was examined using the analysis of covariance structure. Goodness of fit indices including the goodness of fit index (GFI), adjusted GFI (AGFI), comparative fit index (CFI) and root mean square error of approximation (RMSEA) were adopted to evaluate the models. We inputted the significant variables found through the above method and used multi‐group covariance structure analysis to compare models of burnout and intent to resign and their associated factors between countries.
3. Missing values were processed using SPSS missing values.

### 3.6 Ethics

The ethics review board of the authors’ affiliate university approved the study (No. 14,021). The nurse manager contacted nurses to request their participation in the study, and participation was voluntary. Each potential subject was informed of the nature of the study in a relevant letter. Nurses were assured of anonymity and the confidentiality of their information. No code numbers or other identifying marks were printed on the questionnaire. The code number was included on the questionnaire when it was returned.

## 4 RESULTS

### 4.1 International comparison of each scale

We conducted a one‐factor analysis of variance for nurses working in hospitals in the United States, Canada, Japan, Thailand and Malaysia using stressors, burnout and intention to leave (Table 1). The Tukey method was used for multiple comparisons. Regarding stressors, the results indicated that there were significant differences with regard to the following: “death and dying” (F [4,1270] = 40.62; *p* <.01), “conflict with physicians” (F [4,1270] = 25.34; *p* <.01), “inadequate preparation” (F [4,1270] ] = 15.47; *p* <.01), “lack of staff support” (F [4,1270] = 17.37; *p* <.01), “conflict with other nurses” (F [4,1270] = 8.93; *p* <.01), “workload” (F [4, 1270] = 92.37; *p* <.01) and “uncertainty concerning treatment” (F [4, 1270] = 27.57; *p* <.01). For burnout, there were significant differences in “EX” (F [4,1270] = 99.87; *p* <.01), “CY” (F [4,1270] = 12.64; *p* <.01) and “PE” (F [4,1270] = 251.41; *p* <.01). In terms of intention to leave, there were significant differences in “I plan to leave my hospital as soon as possible” (F [4,1270] = 69.59; *p* <.01), and “I plan to stay in my hospital as long as possible” (F [4,1270] = 17.79; *p* <.01). As a result of multiple comparisons, the following were the country‐wise standings for stressors: for “death and dying,” Canada and Malaysia scored highest (*p* <.01), for “conflict with physicians,” Malaysia and Canada scored highest and Thailand lowest (*p* <.01), for “inadequate preparation,” Japan scored highest and Thailand lowest (*p* <.01), for “lack of staff support,” Japan scored lowest (*p* <.01), for “conflict with other nurses,” Malaysia scored highest (*p* <.01), for “workload,” Japan scored highest (*p* <.01), and for “uncertainty concerning treatment,” Japan and Malaysia scored highest (*p* <.01). Regarding the aspects of burnout, Japan scored highest for EX (*p* <.01), Malaysia scored highest for CY (*p* <.01), and Japan scored lowest for PE (*p* <.01). Regarding intention to leave or stay, Japan and Malaysia scored highest on “I plan to leave my hospital as soon as possible” (*p* <.01), and Thailand scored highest on “I plan to stay in my hospital as long as possible” (*p* <.01).

**TABLE 1:** International comparison of each scale

	Canada		Japan		Malaysia		Thailand		US		F	P
	M	*SD*	M	*SD*	M	*SD*	M	*SD*	M	*SD*		
Death and dying	11.00	3.90	8.68	3.63	10.81	3.72	7.33	3.56	9.94	4.22	40.62	**
Conflict with physicians	6.43	2.77	5.92	2.51	6.74	2.44	4.51	2.06	5.82	2.88	25.34	**
Inadequate preparation	3.37	1.89	4.01	1.72	3.76	1.67	2.95	1.39	3.24	1.75	15.47	**
Lack of staff support	3.30	1.67	2.78	1.63	3.92	1.63	3.27	1.43	3.33	1.67	17.37	**
Conflict with other nurses	5.81	2.85	5.01	3.20	6.08	2.71	4.82	2.11	5.40	2.85	8.93	**
Workload	5.63	2.80	9.52	3.33	7.02	3.37	4.95	3.13	5.93	3.08	92.37	**
Uncertainty concerning treatment	4.09	2.77	5.57	2.60	5.68	2.89	3.93	2.23	4.10	2.72	27.57	**
Exhaustion	18.28	6.06	22.35	6.10	15.92	7.12	11.59	5.12	17.11	6.83	99.87	**
Cynicism	13.23	6.82	12.99	7.56	14.82	6.17	10.42	4.34	12.95	7.27	12.64	**
Professional efficacy	28.43	4.94	14.63	7.11	24.94	6.13	26.09	6.78	27.38	5.02	251.41	**
Leave my hospital	2.49	1.12	3.44	1.06	3.47	1.64	1.95	0.89	2.69	1.39	69.59	**
Stay in my hospital	3.38	1.17	3.14	1.13	3.19	1.20	3.89	1.15	3.03	1.35	17.79	**

** *p* <.01

### 4.2 Consideration of the hypothetical model in the simultaneous multi‐population analysis of covariance structure

**FIGURE 2:**
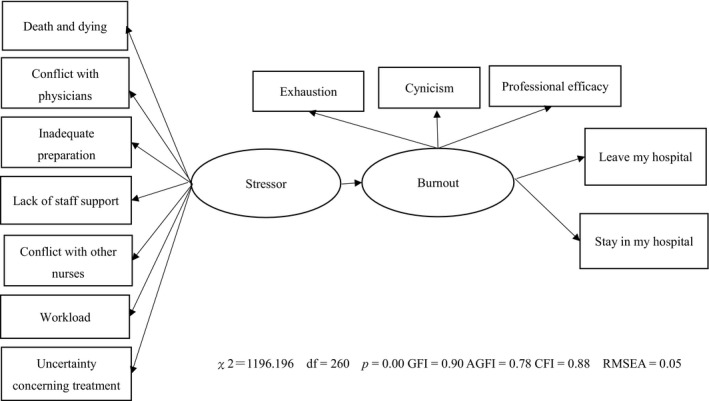
Final adopted model

The hypothetical model was examined using the analysis of covariance structure. Goodness of fit indices including the GFI, adjusted GFI (AGFI), CFI and RMSEA were adopted to evaluate the models. We obtained of model from stressor ⇒ burnout ⇒ turnover intention (χ^2^ =1,196.196, *df* =260, *p* =.00, GFI =0.90 AGFI =0.78, CFI =0.88, RMSEA =0.05; Table 2; Figure 2).

**TABLE 2:** Consideration of the hypothesis model in the simultaneous multi‐population analysis of covariance structure

			Canada	Japan	Malaysia	Thailand	US
			**path** coefficient	**path** coefficient	**path** coefficient	**path** coefficient	**path** coefficient
Burnout	<‐‐‐	Stressor	0.48**	0.46**	0.54**	0.76**	0.73**
Death and dying	<‐‐‐	Stressor	0.77**	0.70**	0.76**	0.86**	0.86**
Conflict with physicians	<‐‐‐	Stressor	0.75**	0.81**	0.79**	0.81**	0.81**
Inadequate preparation	<‐‐‐	Stressor	0.83**	0.66**	0.86**	0.68**	0.83**
Lack of staff support	<‐‐‐	Stressor	0.73**	0.52**	0.79**	0.76**	0.75**
Conflict with other nurses	<‐‐‐	Stressor	0.84**	0.59**	0.86**	0.84**	0.79**
Workload	<‐‐‐	Stressor	0.71**	0.70**	0.82**	0.85**	0.79**
Uncertainty concerning treatment	<‐‐‐	Stressor	0.74**	0.82**	0.82**	0.68**	0.73**
Exhaustion	<‐‐‐	Burnout	0.88**	0.93**	0.74**	0.84**	0.83**
Cynicism	<‐‐‐	Burnout	0.96**	0.79**	0.99**	0.92**	0.89**
Professional efficacy	<‐‐‐	Burnout	−0.37**	−0.24**	−0.31**	−0.23*	−0.39**
Leave my hospital	<‐‐‐	Burnout	0.48**	0.42**	0.28**	0.40**	0.61**
Stay in my hospital	<‐‐‐	Burnout	−0.46**	−0.28**	−0.13*	−0.29**	−0.50**

Abbreviation: *n*.s, nonsignificant.

* *p*<.05

** *p*<.001

Therefore, the hypothetical model was verified (Maslach et al., 1996). When we tested the difference between the parameters, we found that the path coefficient from stressor ⇒ burnout was the highest for Thailand (*p* <.05). Further, the path coefficient from burnout ⇒ stay in my hospital was the highest for the US (*p* <.05).

The results indicate that the model fit is adequate for explaining the associations between stressors, burnout and turnover intention. In other words, in the five countries, reducing stressors could reduce burnout and turnover.

### 4.3 Demographic information of each country

The demographic factors are presented in Table 3. Missing values were processed using SPSS missing values (Table 3).

**TABLE 3:** Demographic information of each country

		country									
		Canada		Japan		Malaysia		Thailand		US	
		*N*	%	*N*	%	*N*	%	*N*	%	*N*	%
Age	20–24	13	4.2%	56	17.7%	4	1.7%	6	2.8%	9	4.6%
	25–29	64	20.6%	64	20.2%	22	9.3%	45	21.3%	56	28.9%
	30–34	90	29.0%	57	18.0%	23	9.7%	53	25.1%	30	15.5%
	35–39	0	0.0%	56	17.7%	36	15.2%	43	20.4%	17	8.8%
	40–44	45	14.5%	33	10.4%	52	21.9%	35	16.6%	9	4.6%
	45–49	31	10.0%	27	8.5%	37	15.6%	17	8.1%	15	7.7%
	50–54	28	9.0%	17	5.4%	39	16.5%	9	4.3%	22	11.3%
	55+	39	12.6%	7	2.2%	24	10.1%	3	1.4%	36	18.6%
Gender	Male	25	8.2%	18	5.7%	6	2.5%	13	6.2%	14	7.2%
	Female	281	91.8%	300	94.3%	230	97.0%	196	93.8%	180	92.8%
Marital status	Single	113	36.7%	177	56.0%	28	11.8%	77	36.7%	88	45.4%
	Married	195	63.3%	139	44.0%	207	87.3%	129	61.4%	106	54.6%
What is your highest degree in nursing?	Diploma of nursing	83	27.3%	287	90.3%	162	66.9%	4	1.9%	3	1.5%
	Bachelor in nursing	188	61.8%	26	8.2%	61	25.2%	195	92.4%	124	63.9%
	Master of nursing	13	4.3%	0	0.0%	7	2.9%	11	5.2%	62	32.0%
	Doctorate in nursing	0	0.0%	0	0.0%	0	0.0%	0	0.0%	3	1.5%
	Others	20	6.6%	5	1.6%	12	5.0%	1	0.5%	2	1.0%
What is your employment status at your current job?	Full‐time	308	99.7%	309	97.5%	242	100.0%	209	99.5%	164	84.5%
	Part‐time	1	0.3%	7	2.2%	0	0.0%	1	0.5%	18	9.3%
	Casual/Various	0	0.0%	1	0.3%	0	0.0%	0	0.0%	12	6.2%
What type of licence do you have?	Registered Nurse	179	58.5%	297	98.0%	188	78.3%	202	96.7%	180	92.8%
	Licence Practice Nurse	74	24.2%	2	0.7%	11	4.6%	0	0.0%	1	0.5%
	Public Health Nurse	53	17.3%	0	0.0%	8	3.3%	0	0.0%	0	0.0%
	Midwife	0	0.0%	3	1.0%	29	12.1%	2	1.0%	0	0.0%
	Advance Practice Nurse	0	0.0%	1	0.3%	4	1.7%	5	2.4%	13	6.7%
Primary area where you work	Critical Care ICU	35	11.4%	15	4.7%	21	8.8%	19	9.0%	2	1.0%
	Critical Care CCU	38	12.4%	0	0.0%	3	1.3%	1	0.5%	24	12.4%
	Emergency	32	10.5%	4	1.3%	7	2.9%	12	5.7%	25	12.9%
	Medical Unit	72	23.5%	109	34.3%	20	8.4%	32	15.2%	52	26.8%
	Surgical Unit	40	13.1%	69	21.7%	24	10.0%	25	11.8%	5	2.6%
	Obstetrical/Family Birthing Unit	15	4.9%	13	4.1%	40	16.7%	19	9.0%	4	2.1%
	Paediatrics Unit	12	3.9%	9	2.8%	14	5.9%	12	5.7%	9	4.6%
	Operating Room	15	4.9%	17	5.3%	14	5.9%	11	5.2%	8	4.1%
	Orthopaedics Unit	12	3.9%	26	8.2%	16	6.7%	0	0.0%	5	2.6%
	Administration	8	2.6%	6	1.9%	9	3.8%	1	0.5%	8	4.1%
	Infection Control	0	0.0%	0	0.0%	3	1.3%	0	0.0%	0	0.0%
	Mental Health/Psychiatric Unit	3	1.0%	0	0.0%	0	0.0%	0	0.0%	6	3.1%
	Burn Unit	18	5.9%	0	0.0%	2	0.8%	0	0.0%	36	18.6%
	Outpatient Clinic	5	1.6%	5	1.6%	8	3.3%	56	26.5%	9	4.6%
	Others	1	0.3%	45	14.2%	58	24.3%	23	10.9%	1	0.5%
How many days off for personal pleasure/activities did you have in last or previous month	None	52	17.0%	16	5.0%	41	17.7%	6	2.9%	39	20.1%
	1 – 3 days	45	14.7%	143	45.0%	126	54.5%	48	23.1%	53	27.3%
	4 – 6 days	53	17.3%	122	38.4%	46	19.9%	67	32.2%	31	16.0%
	7 – 10 days	70	22.9%	25	7.9%	15	6.5%	81	38.9%	40	20.6%
	> 10 days	86	28.1%	12	3.8%	3	1.3%	6	2.9%	31	16.0%
Were you absent from work last year due to health reasons?	Yes	198	64.7%	262	82.1%	50	21.5%	46	22.0%	96	49.5%
	No	108	35.3%	57	17.9%	183	78.5%	163	78.0%	98	50.5%
How many hours of overtime hours did you work in the last month	None	89	29.1%	4	1.3%	92	39.0%	41	19.4%	45	23.2%
	< 5 hr	207	67.6%	55	17.6%	43	18.2%	10	4.7%	41	21.1%
	5 – 10 hr	7	2.3%	81	25.9%	54	22.9%	23	10.9%	44	22.7%
	10 – 20 hr	2	0.7%	84	26.8%	25	10.6%	12	5.7%	37	19.1%
	20 – 30 hr	0	0.0%	32	10.2%	6	2.5%	34	16.1%	16	8.2%
	30 – 40 hr	1	0.3%	15	4.8%	6	2.5%	26	12.3%	5	2.6%
	40 – 50 hr	0	0.0%	12	3.8%	4	1.7%	21	10.0%	5	2.6%
	50 – 60 hr	0	0.0%	12	3.8%	5	2.1%	8	3.8%	1	0.5%
	60 – 70 hr	0	0.0%	5	1.6%	0	0.0%	9	4.3%	0	0.0%
	> 70 hr	0	0.0%	13	4.2%	1	0.4%	27	12.8%	0	0.0%

The sample was derived from Canada (*n* = 309), Japan (*n* = 319), Malaysia (*n* = 242), Thailand (*n* = 211) and the United States (*n* = 194). First, in terms of basic attributes, Japanese nurses were much younger than their counterparts from the other countries, ranging in age from 20 to 24 years (17.7%). Nurses from the United States, Canada, Thailand and Malaysia were of similar, older ages.

Most of the participants were female (Canada: 91.8%, Japan: 94.3%, Malaysia: 97.0%, Thailand: 93.8% and United States: 92.8%). In terms of qualification, “diploma in nursing” was most common in Japan (90.3%) and Malaysia (66.9%); “bachelor's in nursing” was more common in the United States (63.9%), Canada (61.8%) and Thailand (92.4%); and “master's in nursing” was most common in the United States (32.0%), Canada (4.3%) and Thailand (5.2%). A doctorate in nursing was only held by nurses in the United States. In terms of employment type, full‐time work was the most common across all countries (Canada: 99.7%, Japan: 97.5%, Malaysia: 100.0%, Thailand: 99.5% and United States: 84.5%). When asked “What type of licence do you have?”, “registered nurse” was most commonly reported in all countries. For “primary area where you work,” most nurses across all countries (except Malaysia) worked in a medical or surgical unit. When asked, “How many days off for personal pleasure/activities did you have in the previous month,” nurses in the United States (20.1%) were more likely than those from other countries to answer “none.” Nurses in Malaysia (54.5%) selected “1–3 days” more often than nurses in any other country. Furthermore, Japanese nurses (38.4%) selected “4–6 days” more often than nurses in any other country, while nurses in Thailand (38.9%) tended to report “7–10 days.” Nurses in Canada (28.1%) were mostly likely to report “more than 10 days.”

Japan was the country where the highest proportion of nurses (82.1%) answered “yes” to the question “Were you absent from work last year owing to health reasons?” In the United States, Canada and Japan, most of the respondents answered “yes,” while in Thailand and Malaysia, most of the respondents answered “no.” For “How many overtime hours did you work in the last month?” “none” was the most common response in Malaysia and Canada compared with the other countries. For “five hours or less,” Canadian nurses (67.6%) reported this level more frequently than those in other countries.

In Japan and Thailand, overtime hours tended to be more than in other countries.

## 5 DISCUSSION

In terms of demographic factors, Japanese nurses were the youngest, aged 20–24. In general, working women leave their jobs in their early 30s and return to work in their late 30s and 40s, so the employment rate is known to follow an M‐shaped curve. However, nurses tend not to return to work after leaving their jobs (Ministry of Health Labour and Welfare, 2019b). The tendency for more Japanese nurses to be in their 20s than nurses in other countries is captured by this study. In the international comparison, the score for “I plan to leave my hospital as soon as possible” was highest in Japan and Malaysia. Both countries were also characterized by a significant number of nurses holding a diploma in nursing. It has been pointed out that there is a high tendency towards turnover among nurses who obtain their education from training schools (Suzuki et al., 2006). The fact that a significant number of Japanese nurses in this survey had a diploma in nursing may have been a reason for their greater willingness to leave their jobs. Although the popularity of the “bachelor's in nursing” qualification is increasing in Japan, there is still a large percentage of nurses who hold a diploma in nursing. This level of education has been reported to affect not only turnover, but also patient care.

However, while Malaysian and Japanese nurses demonstrated a high trend towards burnout and intention to leave, they provided contradictory responses to “Were you absent from work last year owing to health reasons?” Japan had the highest number of affirmative responses and Malaysia had the highest number of negative responses. It appears that while nurses in Malaysia experienced high burnout and indicated their intention to leave, they continued to work. However, the impact of burnout on turnover in Malaysian nurses tended to be lower than in other countries. In Japan, burnout and intention to leave were high, and the question “Were you absent from work last year owing to health reasons?” received many affirmative responses. The 2011 Hospital Nursing Status Survey of the Japanese Nursing Association reported that the number of nurses who took long‐term sick leave of one month or more in FY 2010 was 7,483, of which 2,669 were full‐time nurses (with a medical certificate) who took leave due to mental health problems (Japanese Nursing Association, 2012). In the present study, the results were similar and more pronounced in comparison to other countries. One of the reasons for this may relate to the high number of overtime hours, as the results indicated that nurses in Japan and Thailand worked the most overtime hours (Nantsupawat et al., 2014; Sawaengdee, 2017).

In addition, Japanese nurses experienced a greater load of stressors than those in other countries, indicating a sizable nursing workload. In Japan, there are active policies aimed at realizing a work‐life balance, such as those recommended by the Ministry of Health, Labour and Welfare’s (2013) “Project Team on Improvement of Employment Quality of Nurses”—which was aimed at retaining and promoting nurses—and the Japanese Nursing Association's “Project to Promote Employment of Nurses in Various Work Styles.” Reducing nurses’ workload may lead to the prevention of turnover. In addition, there is a need to promote stress management interventions, such as cognitive‐behavioural therapy, which has been empirically supported by Ohue et al., (2015).

In the United States, the professional effects of burnout tended to be the highest. To combat this, the country has made considerable progress towards the improvement of nursing conditions. Currently, the Nursing Practice Act allows for an expanded role for nurses, and the American Nurses Association is working to improve the status of professional nurses. The development of doctoral programmes in nursing at major universities has increased the number of qualified and dedicated nursing leaders across the United States (Schwirian, 1998). Total professionalism scores were significantly higher in the United States. Average scores on the professionalism subscale were also significantly higher in the categories of educational preparation, community service, theory development, self‐regulation and autonomy in the United States, whereas publication and communication and research and development were significantly higher in Japan (Tanaka et al., 2015). In the United States, it may be possible to reduce burnout and turnover by building support among nurses through more fulfilling communication. The same seems to be true of Canada. Stressor and burnout reduction are also likely to prevent turnover.

Furthermore, while burnout was low among nurses employed in Thailand, there was a shortage of nurses in hospitals (Sawaengdee, 2017; Thailand Nursing and Midwifery Council., 2016). An analysis of the nursing workforce based on geographic information system surveys and overtime pay revealed a shortage of nurses, with vacancies of 15%–26% of all nursing positions in public hospitals (Thailand Nursing and Midwifery Council., 2016). Regarding nurse burnout in Thailand, 32% of nurses reported high levels of mental fatigue, 18% reported high levels of separation, and 35% reported low levels of personal achievement (Nantsupawat et al., 2016). Owing to the serious shortage of nurses, the number of overtime hours worked by Thai nurses in the previous month was as high as in Japan. In this context, it is considered that a stable social status is the reason why Thai nurses are less likely to burn out or leave their jobs. However, the path coefficient from stressor ⇒ burnout was higher than for the other countries. In other words, stressors are a prominent factor in Thai nurses’ burnout. Thailand may also be characterized by low “workload” scores, despite having the same high number of overtime hours as Japan. Steger (2003) explains that Japanese people are unusually committed to “working hard” and that the effort itself is more highly valued than the results (Kaoru, 1987). Thus, these Japanese cultural behavioural patterns may have a significant impact. Additionally, Thai people tend to avoid conflict (Ramitanon, 2007). In this study, this tendency may have been responsible for their low levels of conflict with physicians and other nurses. In the analysis of Malaysia, “diploma in nursing” was the most common qualification, “cynicism” in burnout was higher than other countries, and the score of “I plan to leave my hospital as soon as possible” was high. In Malaysia, public and private hospitals are treated differently; this report focuses on private hospitals, which offer better treatment. Nevertheless, “CY” and “I plan to leave my hospital as soon as possible” scores were high. Considering the stressors, “conflict with physicians,” “lack of staff support,” “conflict with other nurses,” “workload” and “uncertainty concerning treatment” tended to be higher. In other words, relationships in the workplace may play a major role. Improved relationships may help prevent burnout and reduce turnover intention. Given this study's results, it is necessary to prevent turnover to solve the problem of nursing shortage in all countries. Therefore, we believe that environmental adjustments (Spooner‐Lane & Patton, 2007) and interventions targeting individual perceptions (Ohue et al., 2011, 2015) are needed.

As explained above, compared to other countries, burnout “exhaustion” was the highest in Japan and “cynicism” and intention to leave the job were the highest in Malaysia. Thailand had lower burnouts and turnover than other countries and higher professional efficacy than the United States and Canada. In all countries, reducing stressors is important for reducing burnout and intention to leave jobs, especially as they relate to “lack of support.”

It should be noted that this study was conducted before the start of the COVID‐19 pandemic. A comparative study of various factors related to burnout and intention to leave a job in each country in the event of an emergency, such as current circumstances, suggests the importance of a support system that is tackled on a daily basis.

### 5.1 Limitations

One key limitation of this study is that the cross‐sectional study design limits our ability to establish temporal relationships and ascertain causality. Furthermore, nurses reporting on burnout and intention to leave their own hospital work settings based on social desirability bias could affect the results. Nurse managers being the ones to select study participants potentially introduce additional bias. Moreover, the study is limited by its use of convenience sampling. For example, the North American countries are very diverse, both geographically and culturally. Therefore, as the data were collected from only one area in each country, the results are not widely generalizable. This has implications for future studies with additional sites. The ethics approval for each study site also varied immensely between the countries. The required process for the developed countries was more stringent and complex, taking much longer to complete than in the developing countries. This may have had sampling implications. The Canadian nurses in this study live in a province that requires the “Bachelor of Science in nursing” degree for entry to practice the profession, which may also account for some of the differences in the data. Further studies with larger samples recruited randomly are required.

## 6 CONCLUSION

In this study, an international comparison of stressors, burnout and turnover intention was made among nurses in Canada, Japan, Malaysia, Thailand and the United States. The model—consisting of stressors, burnout and turnover intention—demonstrated a reasonable fit, indicating that in any country, reducing stressors can prevent burnout and reduce turnover. In addition, Japan has nurses who are younger, work more overtime, and have higher burnout and turnover intention. Interventions to address burnout and turnover intention are warranted and should be specific to the related factors in every country. Global interventions are not recommended, as the factors associated with burnout and intent to leave differ among the five countries in this study, and presumably in other countries as well.

## CONFLICT OF INTEREST

No conflict of interest has been declared by the authors.

## AUTHOR CONTRIBUTIONS

Contributor Takashi Ohue was responsible for the organization and coordination of the trial. Takashi Ohue was the chief investigator and also responsible for the data analysis. Takashi Ohue, Laura Bourdeanu, Arlene Pericak, Jean N. Church, Jaruwan Kownaklai, Amorn Suwannimitr, Supaporn Aryamuang and Hamidah Hassan all investigated in their own country. All authors contributed to the writing of the final manuscript. All members of the Study Team contributed to the management or administration of the study.

## Data Availability

The data used in this study can be obtained from the corresponding author upon reasonable request.
